# Retrospective Detection and Phylogenetic Analysis of Cachavirus-Related Parvoviruses in Dogs in China

**DOI:** 10.1155/2023/7010191

**Published:** 2023-03-27

**Authors:** Jun Ji, Qiang Liu, Shunshun Pan, Wen Hu, Xin Xu, Yunchao Kan, Qingmei Xie, Lunguang Yao

**Affiliations:** ^1^Henan Provincial Engineering Laboratory of Insects Bio-Reactor, Henan Provincial Engineering and Technology Center of Health Products for Livestock and Poultry, Henan Provincial Engineering and Technology Center of Animal Disease Diagnosis and Integrated Control, Nanyang Normal University, Nanyang 473061, China; ^2^Zhongjing Research and Industrialization Institute of Chinese Medicine, Zhongguancun Scientific Park, Meixi, Nanyang, Henan 473006, China; ^3^College of Animal Science, South China Agricultural University, Guangzhou 510642, China

## Abstract

Cachavirus (CachaV) infection was first reported in the USA in 2019. This virus has been previously detected in pet dogs and cats in China. In the present study, we retrospectively examined this virus in 413 dogs and 127 cats. Swab samples obtained from these animals were collected during 2015–2017. Notably, CachaV was detected in four samples from dogs with diarrhea but not in cats; however, the correlation between healthy dogs and those with enteritis was not statistically significant. Furthermore, we amplified early complete genomic sequences of the four strains detected in our study dogs (CHN1601, CHN1602, CHN1703, and CHN1704). Among these strains, the sequence identity of the NS1 protein and the seven previously reported strains in China were 97.44%–99.7%, whereas that of VP1 protein was 98.02%–99.6%. Interestingly, in the NS1 coding region, CHN1704 demonstrated 99.7% (highest) similarity with the CachaV strain NWT-W88 detected from a wolf and 64.5% similarity with the NS1 of a bat parvovirus (BtPV) strain. Conversely, in the VP1 coding region, CHN1703 demonstrated 99.7% (highest) similarity with the prototype CachaV strain IDEXX1 detected from dogs and 63.3% similarity with BtPV strain. For the phylogenetic analysis of NS1 and VP1, the four strains detected during 2016-2017 were merged with other Chinese and foreign CachaV strains to form the major branch. We believe that these results helped improve the understanding of how CachaV evolved and suggest that the virus has been circulating in China since at least March 2016.

## 1. Introduction

Parvovirus is a small linear nonenveloped virus with single-stranded 5-6-kilobase-long DNA genomes [1]. At present, 26 genera have been identified and categorized under the *Parvoviridae* family by the International Committee on Taxonomy of Viruses [1]. A recent study proposed a reorganization of the *Parvoviridae* family with the creation of a third subfamily *Hamaparvovirinae* to include both invertebrate and vertebrate viruses [2]. This subfamily would contain viruses that were previously labeled as unclassified chapparvoviruses, namely, *Hepanhamaparvovirus*, Penstyrhamaparvovirus, *Brevihamaparvovirus*, *Ichthamaparvovirus*, and *Chaphamaparvovirus* [3].

At present, the recently identified genus *Chaphamaparvovirus* comprises rat parvovirus 2 [4], common vampire bat (*Desmodus rotundus*) parvovirus [2], murine kidney parvovirus [5], murine chapparvovirus [6], turkey parvovirus 2 [7], porcine parvovirus 7 [8], chicken chapparvovirus 1 and 2 [9], Cachavirus (CachaV) [10], and Fechavirus [3]. Notably, CachaV was first detected in 2019 in dog feces in the USA, raising concerns about the emergence of this novel parvovirus in dogs. This virus is composed of two open reading frames (ORFs), which encode NS1 and VP1, respectively [10]. In our previous study, anal swabs of dogs and cats investigated in China in 2018-2019 were found to be positive for CachaV [11, 12].

In the present study, we traced DNA from dog anal swabs collected during 2015–2017 in order to assess the spread of CachaV in China.

## 2. Materials and Methods

### 2.1. Sample Collection and DNA/RNA Extraction

For this study, anal swab samples of 413 dogs (139 healthy and 274 with diarrhea) and 127 cats (31 healthy and 96 with diarrhea) were collected from pet clinics and hospitals in the provinces of Henan, Hubei, and Zhejiang in China between March 2015 and December 2017. The collected swab samples were washed with 1 mL of phosphate-buffered saline before being subjected to viral nucleic extraction using the EasyPure^®^ Viral DNA/RNA Kit (TransGen Biotechnology, Inc., Beijing, China) according to the manufacturer's protocols. The extracted DNA and RNA were then stored at −80°C until further use.

### 2.2. Virus Screening

The DNA/RNA extracted from the rectal swabs of dogs were tested for CachaV via nested polymerase chain reaction (PCR) (outer F: 5′-CAACTAGCCGAATGCAGGGA-3′, outer-R: 5′-CGATAACATCCCCGGACTGG-3′ for the first round of amplification, and inner-F: 5′-AGCTCAGTTTGGCCCAGATC-3′, inner-R: 5′-AGAGGGATCGCTGGATCTGT-3′ for the second round of amplification) [10], canine coronavirus (F: 5′-CACTAAACTCAAAATGTTGATTC-3′ and R: 5′-TTAAGGATTAAAAACATATTCTA-3′) [13], canine distemper virus (F: 5′-AGCTAGTTTCATCTTAACTATCAAATT-3′ and R: 5′-TTAACTCTCCAGAAAACTCATGC-3′) [14], canine rotavirus (F: 5′-GACGGVGCRACTACATGGT-3′ and R: 5′-GTCCAATTCATNCCTGGTGG-3′) [15], canine bufavirus (F: 5′-CTGGTTTAATCCAGCAGACT-3′ and R: 5′-TGAAGACCAAGGTAGTAGGT-3′) [16], and canine parvovirus (F: 5′-AGAGACAATCTTGCACCAAT-3′ and R: 5′-ATGTTAATATAATTTTCTAGGTGCT-3′) [17] via PCR/reverse transcription-PCR, as previously described. Furthermore, to quantify viral load of CachaV in fecal samples, quantitative PCR (qPCR) was performed using perfect-Start™ probe qPCR SuperMix (TransGen Biotech, Beijing, China) and Bio-Rad Laboratories CFX96 real-time fluorescent quantitative PCR System (Shanghai, China), with the primer pair (F: 5r TCTGGATTTTCTTCTATTGTA-3TGGATTTTCTTCGGATGTGGAAAGTGTTTA-3A) and probe (5prFAM/AAGCAATKCGTTCAAGTGACCATCC/BHQ1-3T) targeting the NS1 gene of CachaV. Notably, the real-time qPCR reaction system for each reaction included 10 *μ*L of 2× perfect-Start probe qPCR SuperMix, 0.2 *μ*M of forward and reverse primer, 0.2 *μ*M of prob, and 1 *μ*g template DNA or 1 *μ*L diluted standard DNA (the prepared CachaV-NS1 gene plasmid). The reaction was conducted as follows: 95°C for 5 min, 40 cycles at 95°C for 10 s, and 60°C for 30 s. A standard curve was plotted based on the results of parallel PCRs performed on ten-fold serial dilutions (10^7^–10^2^ copies per reaction) of standard DNA. Quantities of absolute DNA of CachaV in fecal samples were calculated via normalization to the standard curve, and these quantities were presented as the means of the quantities measured in three repeated reactions per sample. Furthermore, the infection status of the dogs was classified and visualized using the UpSet plot packages in the TBtools software [18].

### 2.3. Amplification, Cloning, and Sequencing of CachaV Genomes

Overlapping gene segments were amplified via PCR using 20 *μ*L reaction mixture containing DNA template (>100 ng/*μ*L), 6 pmol of upstream/downstream primers as previously described [12], PrimeSTAR HS DNA polymerase, and a compatible reaction buffer (TaKaRa Biotechnology Co., Ltd., Dalian, China). The cycling conditions were as follows: initial denaturation at 95°C for 3 min; 30 cycles of denaturation at 95°C for 30 s, annealing at 55°C for 30 s, extension at 72°C for 1 min; and final extension at 72°C for 10 min. The obtained amplicons were then cloned into pMD18-T Easy Vector (TaKaRa Biotechnology Co., Ltd.) for subsequent sequencing (Syn-Biotechnology, Suzhou, China).

### 2.4. Sequence Alignment, Recombination Analyses, and Phylogenetic Analysis

Genome sequences of the obtained strains were assembled and aligned with the reference strains using DNAStar version 7.0 (DNASTAR Inc., Madison, WI, USA). The detailed information of each reference strain is presented in Supplement Table 1. Furthermore, the nearly complete genomes were screened for the presence of chimeric sequences using the methods (RDP, Chimaera, BoosScan, 3Seq, GENECONV, MaxChi, SiScan, and LARD) included in the RDP 4.83 software package [19]. Moreover, recombinations were also predicted using SimPlot, version 3.5.1. Phylogenetic tree analysis was conducted based on genome nucleotide sequence, the deduced amino acid (aa) sequences of NS1 and VP1 proteins of the four strains as well as the reference strains using maximum likelihood estimation in the MEGA X software with 1000 bootstrap replicates [20]. The phylogenetic trees were annotated and displayed using an online software (https://www.chiplot.online/).

### 2.5. Protein Mutation in NS1 and VP1

MegAlign was used to compare the sequences of NS1 and VP1 of the four strains identified in this study with those of the seven reference CachaV strains (two from USA, five from China) identified from dogs; the representative amino acid mutations in the four strains were counted and summarized. The altered amino acid sequences were modeled in SWISS-MODEL (https://swissmodel.expasy.org/interactive) according to varying amino acid sites. Subsequently, Protein Data Bank files were created using PyMOL for collation and preservation.

## 3. Results

### 3.1. Molecular Investigations

**Figure 1:**
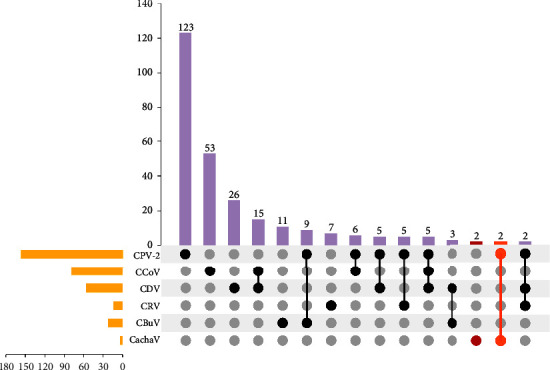
Positive detection rates for CachaV, CPV-2, CCoV, CDV, CRV, and CBuV. Infection caused by the combination of the six pathogens illustrated using an UpSet plot. The UpSet plot presents the distribution of different viruses in the identified samples. The bar chart above represents the number of genes identified in each group. The bar chart at the bottom left represents the number of positive results for each type of pathogen. The dotted line at the bottom right presents the types of events identified in each group. CachaV, cachavirus; CPV-2, canine parvovirus-2; CCoV, canine coronavirus; CDV, canine distemper virus; CRV, canine rotavirus; CBuV, canine bufavirus.

Among the samples obtained from Henan, Hubei, and Zhejiang in 2016-2017, CachaV infection and gastrointestinal disease were not significantly associated based on the results of the chi-square test (*P*=0.3052) after the fractions of nested PCR-positive fecal samples were compared between healthy dogs (0/139, 0% positive) and those with diarrhea (4/274, 1.50%). Meanwhile, the qPCR assay revealed 100% agreement with the nested-PCR assay; viral DNA copies in the viral DNAs extracted from the four positive samples are shown in Table 1. The infection status of the dogs is presented in Figure 1, and the clinical histories of the four infected dogs are shown in Table 1.

### 3.2. Sequence Analysis and Recombination Detection

The genome sequences of the four Chinese CachaV strains (CHN1601, CHN1602, CHN1703, and CHN1704) identified in this study were amplified using four specific primers (517–4123 bp according to the CachaV strain, IDEXX1; accession number: MH893826). Notably, the genomic structures of these four strains were consistent with that of IDEXX1, with two major ORFs encoding NS1 (663 aa) and VP1 (504 aa). Moreover, similar to IDEXX1 and other previously reported Chinese CachaV strains, the ATP-binding Walker loop motif GPSNTGKS was present in the NS1 ORF of these strains.

The four CachaV strains identified in this study and the seven previous strains (five from dogs (accession numbers: MT123283–MT123287); two from cats (accession numbers: MN928790-MN928791)) shared 97.44%–99.7% and 98.02%–99.6% identities in NS1 and VP1 sequences, respectively. In addition, NS1 of CHN1704 demonstrated the highest similarity (99.7%) with the NWT-W88 strain identified in a wolf, whereas CHN1703 demonstrated the highest similarity (99.8%) with the prototype strain IDEXX1 detected in dogs. Notably, sequence alignment of these four CachaV strains with *Chaphamaparvovirus* strains identified from other species revealed that NS1 of bat parvovirus strain BtPV is 63.3% similar to CHN1704, and VP1 of BtPV is 64.5% similar to CHN1703. Furthermore, no clear recombination signals were observed among these four Chinese sequences and the reference strains across their entire genomes.

### 3.3. Phylogenetic Analysis of NS1 and VP1

**Figure 2:**
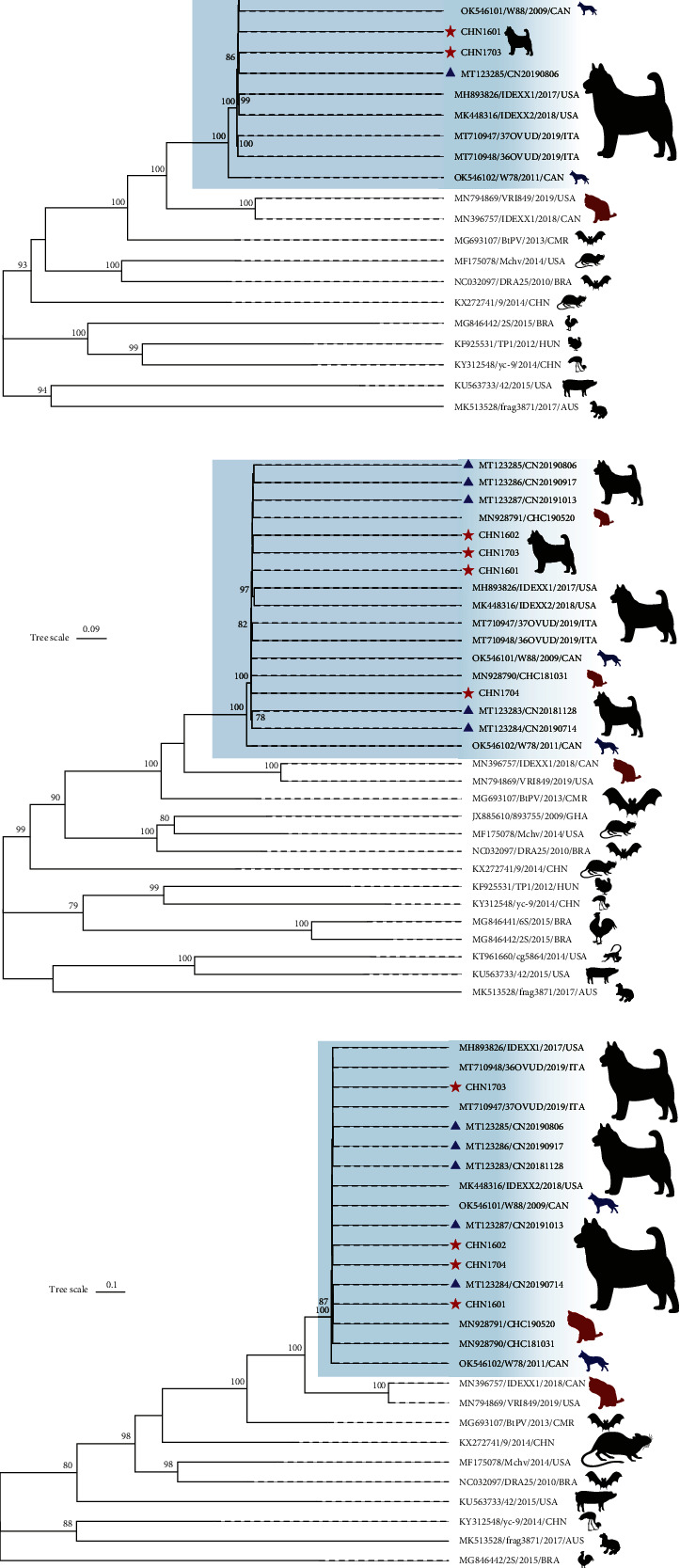
Phylogenetic tree analysis based on nucleotide sequences of the complete genome (a), amino acid sequences of NS1 (b), and VP2 (c) sequences of the identified CachaV strains compared with the sequences of all CachaV and reference strains of representative parvoviruses available in the GenBank. Red asterisks indicate the strains identified in the present study. Blue triangles indicate the reference CachaV strains identified in China.

The genome sequence, NS1 and VP1 sequences from the identified four strains were analyzed using an evolutionary tree (Figure 2). We found that the evolutionary relationship between these four strains was similar to that between the Chinese CachaV strains identified in 2018–2019 and IDEXX1 and IDEXX2 within one branch. VRI849—which was detected in cats from the USA—was highly similar to CachaV grouped into carnivore chapparvovirus 1, clustered with IDEXX1 (feline chapparvovirus strain). Moreover, the presence of feline and NWT-W78 strains (accession number: OK546102, CachaV detected in gray wolf) indicated the complicated classification and evolution of CachaV strains.

### 3.4. Mutation and Structural Analysis of NS1 and VP1

**Figure 3:**
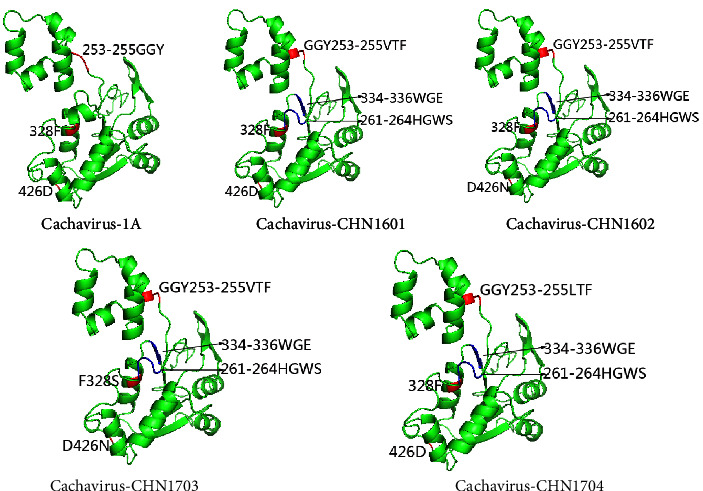
Predicted tertiary structure model of NS1 from different CachaV strains.

We compared the sequences of the identified four strains with those of IDEXX1 and found 10 and 5 representative mutation sites in the NS1 and VP1 proteins, respectively. In NS1, the mutations of Gly254Thr, Tyr255Phe, Gly603Arg, and Arg607Gly were found only in Chinese CachaV strains identified in this study and our previous reports [11, 12]. In contrast, the mutations of Lys622Arg in NS1 and Ala186Phe in VP1 were found only in the strains obtained in this study. Detailed information about mutation sites is presented in Table 2. Mutations associated with changes in the predicted tertiary structure model of NS1 and VP1 are shown in Figure 3.

## 4. Discussion

CachaV infection was first reported in the USA in 2019 in healthy dogs (1.47%), indicating that dogs can act as hosts of this virus [10]. In our previous study conducted in 2018-2019, we collected 85 and 323 swabs from healthy dogs and dogs with diarrhea, respectively. Notably, CachaV strains were detected only in dogs with diarrhea (1.55%) [11]. This virus was previously detected in two Chinese pet cats in a previous study [12]. In the present study, a retrospective test for CachaV was performed, and the positive test rate was found to be 1.46% (4/274), which was also identified only in dogs with diarrhea. This retrospective test could advance the first detection of CachaV in China to 2016.

*Chaphamaparvovirus* strains have been detected in various organisms, including an exceptionally broad range of vertebrates [21]. In this retrospective detection study, the identified virus sequences were the same as CachaV sequences first discovered in China. However, this virus has not been isolated via viral experiments. To date, only mouse kidney parvovirus of the genus *Chaphamaparvovirus* has been isolated from laboratory and wild mouse populations and has been associated with nephritis in both immunocompromised and immunocompetent laboratory mice [22, 23]. This virus can be transmitted via the fecal-oral route, which indicates its potential as a gastrointestinal agent [5].

Similar to a previous report on Chinese CachaV strains detected in dogs and cats, statistical analysis of our study indicated no correlation between the presence of virus and diarrhea (*P* > 0.05), although CachaV strains were detected only in dogs with diarrhea. To further explore the pathogenesis of CachaV, virus isolation and animal experiments are warranted.

The retrospectively detected four strains of CachaV in this study were similar to the seven CachaV strains detected in 2018-2019 based on the sequence identity analysis. Moreover, the phylogenetic tree relationships were examined between CachaV and BtPV [4]. However, to understand the transmission and evolution of CachaV and other *Chaphamaparvoviruses*, persistent investigation and epidemic analyses are required.

Mutation analysis revealed that the modeled structure of NS1 in the four strains differed remarkably from that of NS1 in the IDEXX1 strain, including mutations such as G254T, Y255F, and consecutive mutations at positions 261–264 (HGWS) and 334–336 (WGE). However, the specific mutations that produce the structural changes observed in this protein remain unknown. Combining the Chinese strains detected in dogs and cats in 2018-2019 [11, 12], we observed that the G254T and Y255F mutations in NS1 occurred at both sites, suggesting that these sequences have a common origin and that strains with these mutations are now widespread in the area. To further understand CachaV, the regularity of virus mutations needs to be studied extensively with more data.

## 5. Conclusion

In this study, four CachaV strains were detected in the swab samples of dogs with diarrhea, advancing the first detection of this virus in China to 2016. Phylogenetic and mutational analyses provided important theoretical basis and data to trace CachaV and establish its epidemiology in China.

## Figures and Tables

**Table 1:** Details of the four Chinese dogs infected with cachavirus strains detected in this study.

Sample name	Health status	Age (months)	Province	Date	Coinfection	Viral copy numbers (per *μ*g)
Cachavirus-CHN1601	Diarrhea	8	Henan	March 2016	—	4.31 × 10^5^
Cachavirus-CHN1602	Diarrhea	5	Henan	May 2016	CPV-2	1.56 × 10^5^
Cachavirus-CHN1703	Diarrhea	5	Henan	July 2017	CPV-2	8.79 × 10^4^
Cachavirus-CHN1704	Diarrhea	6	Hubei	October 2017	—	1.06 × 10^5^

**Table 2:** Details of the main amino acid mutation sites in the NS1 and VP1 proteins of cachavirus in Chinese (“CHN-”; this study) compared with reference strain (IDEXX1).

Strains	Substitution of amino acid residues														
	NS1										VP1				
	252	253	254	255	328	426	457	603	607	622	186	265	449	470	493
IDEXX1	S	G	G	Y	F	D	D	G	R	K	A	V	R	K	V
CN20181128	C	L	T	F	F	D	N	R	G	K	A	V	R	R	V
CN20190714	C	L	T	F	F	D	N	R	G	K	A	I	K	K	I
CN20190806	S	V	T	F	F	D	D	R	G	K	A	V	R	K	V
CN20190917	S	V	T	F	F	D	N	R	G	K	A	V	R	K	V
CN20191013	S	V	T	F	F	D	N	R	G	K	A	I	R	K	V
CHC181031	C	L	T	F	F	D	N	R	G	K	A	I	K	K	V
CHC190520	S	V	T	F	F	D	N	R	G	K	A	I	K	K	I
CHN1601	S	V	T	F	F	D	D	R	G	R	A	I	K	K	I
CHN1602	S	V	T	F	F	N	N	R	G	K	P	I	R	K	V
CHN1703	S	V	T	F	S	N	D	R	G	K	A	V	R	K	V
CHN1704	C	L	T	F	F	D	N	R	G	R	P	I	R	R	V

## Data Availability

All data generated or analyzed during this study are included within the article. The datasets used and/or analyzed during the current study are available from the corresponding author upon reasonable request.

## Supplementary Materials

Supplementary Materials Table S1: information about sequences of reference strains used in this study.
