# Supplementary material for: Retrospective Detection and Phylogenetic Analysis of Cachavirus-Related Parvoviruses in Dogs in China
Source: Transbound Emerg Dis. 2023 Mar 27;2023:7010191. doi: 10.1155/2023/7010191 (PMC12017101; doi:10.1155/2023/7010191)
Supplement: Supplementary Materials — Table S1: information about sequences of reference strains used in this study. [file 7010191.f1.docx]

Table S1. Information about sequences of reference strains used in this study

| Strains | Host | Date | Country | Accession Nos. |
| --- | --- | --- | --- | --- |
| CN20181128 | Canine | 28.11.2018 | China | MT123283 |
| CN20190714 | Canine | 14.07.2019 | China | MT123284 |
| CN20190806 | Canine | 06.08.2019 | China | MT123285 |
| CN20190917 | Canine | 17.09.2019 | China | MT123286 |
| CN20191013 | Canine | 13.10.2019 | China | MT123287 |
| CHC181031 | Feline | 31.10.2018 | China | MN928790 |
| CHC190520 | Feline | 20.05.2019 | China | MN928791 |
| 893755 | Eidolon helvum | 03.2009 | Ghana | JX885610 |
| TP1 | Meleagris gallopavo | 03.2009 | Hungary | KF925531 |
| cg5864 | Macaca mulatta | 2014 | USA | KT961660 |
| 42 | Swine | 2015 | USA | KU563733 |
| 9 | Wild rat | 2014 | China | KX272741 |
| yc-9 | Crane | 2014 | China | KY312548 |
| Mchv | Mus musculus | 28.10.2014 | USA | MF175078 |
| BtPV | Eidolon helvum | 2013 | Cameroon | MG693107 |
| 6S | Gallus gallus | 2015 | Brazil | MG846441 |
| 2S | Gallus gallus | 2015 | Brazil | MG846442 |
| IDEXX1 | Canine | 17.12.2017 | USA | MH893826 |
| IDEXX2 | Canine | 09.2018 | USA | MK448316 |
| frag3871 | Sarcophilus harrisii | 2017 | Australia | MK513528 |
| DRA25 | Desmodus rotundus | 23.06.2010 | Brazil | NC032097 |
| IDEXX1 | Feline | 12.2018 | Canada | MN396757 |
| VRI849 | Feline | 29.01.2019 | USA | MN794869 |
| W78 | Canis lupus | 2011 | Canada | OK546102 |
| W88 | Canis lupus | 2009 | Canada | OK546101 |
| 37OVUD | Canine | 2019 | Italy | MT710947 |
| 36OVUD | Canine | 2019 | Italy | MT710948 |
